# Implications of LINC01094 for human malignancies

**DOI:** 10.7717/peerj.20621

**Published:** 2026-01-27

**Authors:** Yan Wang, Yang Yang, Quanying Zhang, Ying Zeng, Yilin Cai, Haiqing Luo, Xiangyong Li

**Affiliations:** 1 Institute of Biochemistry and Molecular Biology, Guangdong Medical University, Zhanjiang, Guangdong, China; 2 Key Laboratory of Liver Injury Diagnosis and Repair and Department of Hepatobiliary Surgery, The 2nd Affiliated Hospital of Guangdong Medical University, Zhanjiang, Guangdong, China; 3 School of Medical Technology, Guangdong Medical University, Dongguan, Guangdong, China; 4 Cancer Hospital of The Affiliated Hospital of Guangdong Medical University, Zhanjiang, Guangdong, China

**Keywords:** Cancers, Clinical characteristics, Growth and metastasis, LINC01094, Molecular mechanisms

## Abstract

Long non-coding RNAs (lncRNAs) constitute a large class of ribonucleic acids, participating in multiple biological events within tumor cells, especially the regulation of transcription. A growing body of literature has revealed that abnormalities of lncRNA expression could result in carcinogenesis and oncogenesis by exerting inhibition or oncogenic effects. LINC01094 is a recently identified lncRNA found to be dys-regulated in an assortment of cancer tissues and control multiple biological processes via competing endogenous RNA (ceRNA) mechanisms. Specifically, LINC01094 functions as a ceRNA to modulate tumor cell growth, invasion, and migration by regulating critical signaling pathways including PI3K/AKT, PTEN/AKT, and Wnt/β-catenin, while also exerting oncogenic effects through transcriptional regulatory networks. Numerous recently published reports have shown that LINC01094 exerts critical functions during the regulation of malignant cell growth, migrating ability, and invasiveness, thereby controlling cancer cell growth and metastasis. In this review, multiple cancer biology functions of LINC01094 documented in published literature are summarized, aiming to inspire innovations in the management of human malignancies under laboratory and clinical settings.

## Introduction

Over the past decades, neoadjuvant therapy (NAT)—encompassing chemotherapy, radiotherapy, and immunotherapy—has revolutionized cancer treatment by improving response rates and surgical outcomes (Yuan et al., 2025). However, substantial challenges persist in enhancing patients’ quality of life, prolonging survival, and mitigating late-stage recurrence. These limitations underscore the urgent need to decipher the molecular mechanisms underlying cancer cell heterogeneity. Therefore, it is crucial to further elucidate the molecular andcellular mechanisms responsible for these unique features of cancerous cells to facilitate improvements in cancer diagnosis and treatment. Recently, the implications of non-coding RNAs (ncRNAs) for carcinogenesis and oncogenesis, as well as the underlying molecular mechanisms, have increasingly attracted research interest. NcRNAs are RNAs that do not encode protein products that exert crucial biological and cancerogenesis-related functions (Anastasiadou, Jacob & Slack, 2018; Esteller, 2011). Generally, ncRNAs are divided into circular RNA and linear RNA according to their structure, and those with a linear structure can be further divided into long ncRNAs (lncRNAs) and microRNAs (miRNAs) (Miano et al., 2021; Yan & Bu, 2021). lncRNAs are a type of ncRNAs composed of more than 200 nucleotides that regulate gene transcription activity through histone modification, chromatin remodeling, interaction with transcription factions, and DNA methylation (Karakas & Ozpolat, 2021; Schmitz, Grote & Herrmann, 2016). Thanks to the advances and improvements in transcriptome analysis methods, as well as the rapid development of bioinformatics, more and more lncRNAs have been discovered recently (St Laurent, Wahlestedt & Kapranov, 2015). More importantly, the dysregulated lncRNA abundance has been tightly associated with the initiation and progression of various malignancies, as well as their invasiveness, metastasis, and drug resistance (Li et al., 2018). For instance, Zhao et al. (2020) demonstrated an inhibition effect of the decreased abundance of lncRNA NEAT1 on the growth, invasiveness, and migrating capability of non-small cell lung cancer cells *via* a miR-204/NUAK1 pathway. Another study found that lncRNA TLNC1 could inhibit the transcription of p53 target genes by interacting with the translocated promoter region (TPR) and facilitate liver cancer metastasis and growth (Yuan et al., 2022a). In addition, several studies have verified that lncRNAs could serve as therapy and prognostic biomarkers for multiple types of malignancies (Bi et al., 2025; Coan et al., 2024; Liu et al., 2023; Shu et al., 2024) .

**Figure 1:**
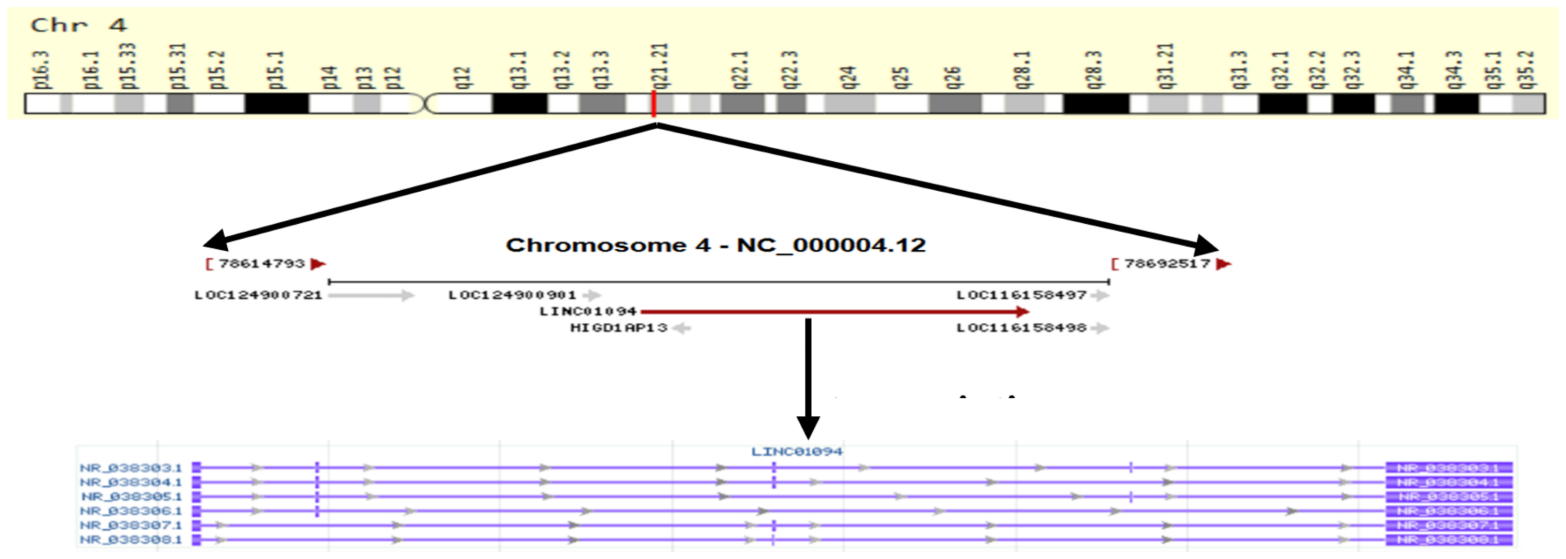
Schematic diagram of the formation of LINC01094. LINC01094 (Gene ID: 100505702; Ensemble: ENSG00000251442), is located on human chromosome 4q21.21. The gene is 52,213 bp in 90 length and composed of 5 exons, which can be transcribed into six transcripts.

**Figure 2:**
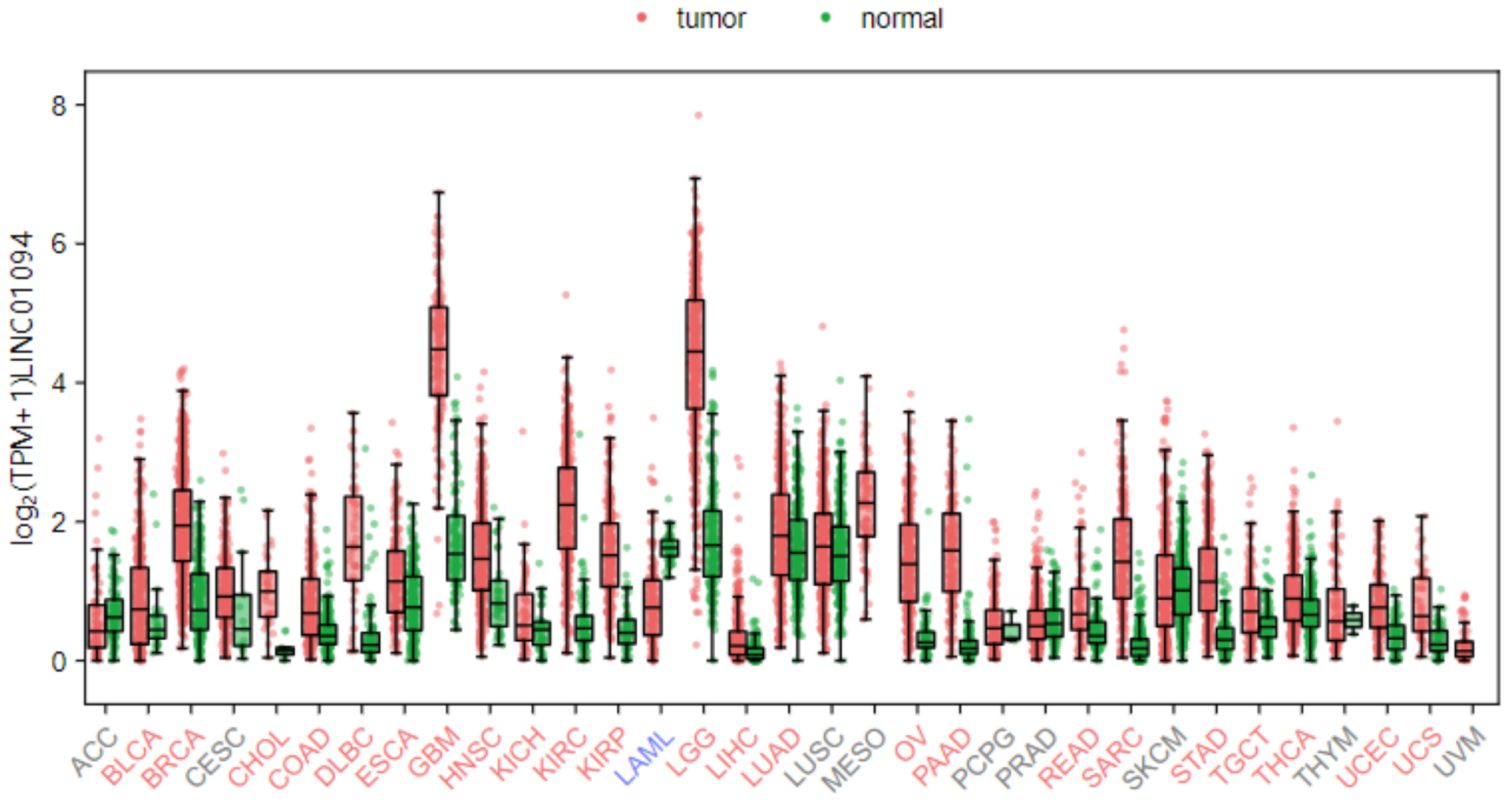
Distribution map of LINC01094 expression in various tumor tissues. Bioinformatics analysis from the GEPIA3 (v1.0) database has revealed that LINC01094 exhibits dysregulated expression across multiple cancer types.

LINC01094 (also called CTEPHA1; Gene ID: 100505702; Ensemble: ENSG00000251442), is a newly discovered lncRNA that located on human chromosome 4q21.21. The gene is 52,213 bp in length and composed of five exons, which can be transcribed into six transcripts (Fig. 1). According to the GEPIA3 (https://gepia3.bioinfoliu.com/express/ediy/#ebox_result) database, the abundance of this lncRNA is abnormally high in various tumor lesions, including clear cell renal cell carcinoma, glioblastoma, and breast carcinoma (Fig. 2). Therefore, several studies have investigated the potential of LINC01094 as a diagnostic biomarker or therapeutic target for multiple malignancies (Xu et al., 2022; Yang et al., 2024; Zhang et al., 2025; Zhou et al., 2022). Herein, we have summarized the regulatory roles or impacts of LINC01094 on diverse cellular processes within tumor cells and the underlying molecular mechanisms. Our review highlights the central role of LINC01094 in molecular mechanistic networks controlling essential biological events within cancerous cells and its promising applicability in the diagnosis and treatment of malignancies.

## Survey Methodology

To ensure the comprehensiveness and completeness, the systematic literature search was conducted across PubMed, Web of Science, and EMBASE databases from January 1, 2000 to June 30, 2025, focusing on human studies investigating the roles of long non-coding RNAs (lncRNAs) in cancer pathogenesis. (1) Search parameters (end date: June 30, 2025; inclusion criteria: human studies only, excluding cell/animal models and non-validation cohorts; duplicates removed *via* automated tools and manual verification; retractions flagged *via* Retraction Watch); (2) study selection flow (now detailed in Fig. S1 with a summary table of included primary studies, specifying model systems and readouts); (3) Fig. 2 documentation (GEPIA3 v1.0, TPM normalization, TCGA tumor cohorts; raw data provided in Table S2); and (4) miRNA target predictions (entered “LINC01094” in the search box of lncRNASNP2 database; selected the “miRNA interaction” module; retrieved high-confidence miRNA candidates (reads per million (RPM) ≥ 1) with binding sites validated by CLIP-seq and sequence complementarity analysis.

## LINC01094 implicated in multiple cancers initiation and progression

Numerous recently published investigations have elucidated the crucial functions of LINC01094 for diverse human cancers. According to their findings, LINC01094 is implicated with the growth and death of cancerous cells, as well as the metastasis, invasiveness, and therapy resistance of malignant lesions. Table 1 summarizes the clinicopathological features of malignancies characterized by high LINC01094 expression.

## Gastric cancer (GC)

Currently, GC remains a major unsolved clinical problem worldwide, resulting in very poor overall survival statistics for affected patients (Joshi & Badgwell, 2021; Rawla & Barsouk, 2018). Therefore, it is important to explore novel biomarkers with high specificity, sensitivity, and efficiency in diagnosing and guiding the targeted therapy for GC.

According to an investigation published in 2021, abnormal LINC01094 expression could be utilized to independently predict GC prognosis (Zhang et al., 2021). Subsequently, other researchers reported that LINC01094 displayed high expression in GC tissues, and its abundance exhibited close association with the clinicopathological characteristics and invasion of GC (Ye et al., 2022). Consistently, evidence from another research also verified the significant overexpression of LIN01094 in GC lesions compared with noncancerous samples and the close correlation of LIN01094 abundance with GC early metastasis and prognosis (Gong et al., 2023). Clinically, LINC01094 was found to contribute to the prognostic prediction and molecular feature assessment in GC patients (Luo et al., 2022; Tu et al., 2022). Interestingly, a recent study used LINC01094 to establish a risk prediction model consisting of seven platelet activation-related lncRNAs in GC, which could provide novel insights for prognostic prediction in GC patients and elucidate the process and mechanism of platelet activation (Yuan et al., 2022b). Furthermore, existing evidence has revealed a close correlation between LINC01094 dysregulation and angiogenesis as well as inflammation in GC tissues, and has shown that it shapes the immunosuppressive tumor microenvironment by sponging miR-17-5p to dually regulate PD-L1 and PD-L2 expression, suggesting that LINC01094 could serve as a novel clinical treatment and prognosis evaluation marker for GC (Xu et al., 2022; Zhang et al., 2024; Zhang et al., 2021). Therefore, further in-depth investigations on the role of LINC01094 as a novel GC biomarker in future studies may effectively improve the early diagnosis rate of malignancy.

**Table 1:** Clinicopathological features of malignancies characterized by high LINC01094 expression.

Cancer type	Sample number	Clinicopathological features analyzed	Prognosis	Refs
Gastric cancer	375	T stage, histological grade, pathological stage,	poor	Gong et al. (2023), Luo et al. (2022), Tu et al. (2022), Ye et al. (2022) and Zhang et al. (2024)
	106	T/N stages		
Glioblastoma	163	Grading tumor diameter KPS score	Poor	Dong et al. (2020), Li & Yu (2020), Liu et al. (2021) and Zhu et al. (2020)
Clear cell renal cell carcinoma	523	Immune cell infiltration radio-resistance	Poor	Jiang et al. (2020a), Jiang et al. (2020b) and Zhou et al. (2022)
Non-small cell lung cancer	483	Metastasis	Poor	Sun et al. (2022) and Wu et al. (2022)
Ovarian cancer	426	FIGO stage overall survival rate	Poor	Xu et al. (2023) and Xu et al. (2020a)
	93	lymph node metastasis		
Pancreatic cancer	91	Tumor size	Poor	Huang et al. (2022) and Luo et al. (2021)
	179	Lymphatic metastasis TNM classification		
Colorectal cancer	122	lymph node metastasis TNM stage	Poor	Zhang et al. (2022)
Breast cancer	1,085	Metastasis	Poor	Wu, Kong & Wu (2021)
	54			

## Glioblastoma (GBM)

GBM is a prevalent malignancy that primarily affects the central nervous system (Lah, Novak & Breznik, 2019). Due to the aggressive lesion growth and drug resistance, conventional therapeutic strategies such as chemotherapy, radiotherapy, and surgery exhibit limited efficacy in managing GBM (McGranahan et al., 2019). As a result, molecular targeted therapy for GBM has increasingly become a research hotspot in recent years. The findings of these studies revealed abnormally high LINC01094 expression in GBM tissues relative to normal controls, suggesting that LINC01094 has a close association with the occurrence and development of GBM (Dong et al., 2020; Liu et al., 2021; Zhu et al., 2020). In addition, research has shown that increased LINC01094 abundance in glioma correlated not only with glioma grading but also with the tumor size and Karnofsky Performance Scale score (Zhu et al., 2020). More importantly, LINC01094 knockdown was found to inhibit the growth and invasive abilities of GBM cells *in vivo* and *in vitro* (Li & Yu, 2020; Zhu et al., 2020). Therefore, it can be inferred that LINC01094 is a crucial lncRNA regulating GBM initiation and progression that can be targeted in therapeutic strategies for GBM.

## Clear cell renal cell carcinoma (ccRCC)

ccRCC, which comprises around four-fifths of renal cell carcinoma cases (Ricketts et al., 2018), is a kind of renal malignant tumor with relatively poor prognosis (Demirjian et al., 2021; Deng et al., 2019). Data from the public TCGA database and the microarray datasets GSE15641, GSE29609, GSE36895, GSE46699, and GSE53757 indicate high LINC01094 expression levels in ccRCC tissues, which were verified through quantitative polymerase chain reaction (PCR) assays comparing the transcript abundance of LINC01094 in ccRCC and matched noncancerous tissues (Jiang et al., 2020a; Xu et al., 2020a). Another clinical study revealed that LINC01094 can affect ccRCC patient prognosis, with LINC01094 upregulation being closely correlated with cancer cell growth, immune cell infiltration, metastasis, and radio-resistance of ccRCC (Zhou et al., 2022). Consistently, Zhang et al. (2024) and Zhang et al. (2022) confirmed that LINC01094 could promote ccRCC radio-resistance by regulating an miR-577/CHEK2/FOXM1 pathway, demonstrating that LINC01094 is potentially a novel therapeutic target for this malignancy (Jiang et al., 2020a). On the contrary, silencing of LINC01094 could inhibit the proliferation, invasiveness, and migrating ability of ccRCC cells through an miR-184/SLC2A3 axis (Xu et al., 2020a). Taken together, these findings indicate that LINC01094 is a valuable molecule that should be focused on in investigations regarding ccRCC neogenesis, progression, and diagnosis, as this will provide novel insights for ccRCC treatment and diagnosis.

## Non-small cell lung cancer (NSCLC)

NSCLC is currently a primary cause of deaths resulting from malignancies worldwide (Duma, Santana-Davila & Molina, 2019). According to previous studies, LNC01094 is remarkably highly expressed in lung squamous cell carcinomas (LUSC) tissues and closely associated with LUSC patient prognosis. Remarkably, analysis of GEO and TCGA databases signified the involvement of LINC01094 in biological events modulating LUSC epithelial to mesenchymal transition (EMT) and necrosis (Sun et al., 2022). Recently, Jiang et al. (2019) found that LINC01094 could promote M2-type macrophage aggregation and lung adenocarcinoma (LUCD) cell metastasis by binding to SPI1 and activating the transcription of the downstream molecule CCL7 (Wu et al., 2022). These findings testify the diagnostic and therapeutic value of LINC01094 for NSCLC.

## Ovarian cancer (OC)

Currently, OC is considered to be a primary cause of female deaths resulting from malignancies worldwide (Armstrong et al., 2021; Ferlay et al., 2021). Due to the anatomical location of primary OC lesions, the early onset of the disease is usually asymptomatic and difficult to diagnose. As a result, many OC patients are diagnosed in an advanced stage, which leads to extensive metastasis, high mortality, and poor prognosis (Torre et al., 2015). LINC01094 has been identified as a pyroptosis-related prognostic marker in ovarian cancer. Importantly, a competitive endogenous inhibition relationship was discovered between LINC01094 and multiple pyroptosis-related genes, including IRF1 and GSDME, and shaped an immunosuppressive tumor microenvironment characterized by the infiltration of macrophages and cancer-associated fibroblasts (Xu et al., 2023). Another study demonstrated that LINC01094 promotes the proliferation, migration, invasion and EMT of OC cells by adsorbing miR-577 (Xu et al., 2020b). In conclusion, LINC01094 has the potential to serve as a prognostic biomarker and treatment target in OC management. Moreover, further clarifying LINC01094’s function during the early stage of OC and its underlying mechanisms will enhance the therapeutic effect in OC patients.

## Pancreatic cancer (PC)

PC, a disease characterized by its notoriously aggressive malignant tumors and a tendency to resist chemotherapy, has a fairly low 5-year overall survival rate (Jiang et al., 2019). PC lesions were reported to express evidently high levels of LINC01094 (Huang et al., 2022), with its high abundance exhibiting correlations with lymphatic metastasis, tumor size, and Tumor-Node-Metastasis (TNM) classification (Luo et al., 2021). Clinically, LINC01094 expression has been proven to independently affect PC patients’ prognosis, with those having higher LINC01094 expression possessing a shorter survival period (Luo et al., 2021). Moreover, *in vitro* and *in vivo* assays showed that upregulation of LINC01094 could enhance the proliferative capacity of PC cells (Luo et al., 2021). In contrast, LINC01094 downregulation significantly reduced the proliferation and metastasis of PC cells (Luo et al., 2021). Furthermore, LINC01094 was also observed to exert a crucial function in promoting PC progression *via* activating the PI3K/AKT pathway (Luo et al., 2021). In conclusion, LINC01094 is implicated with the initiation and progression of PC and can serve as a diagnostic biomarker and treatment target for the malignancy. Notably, the role of LINC01094 in promoting PC development and metastasis may depend on its role in increasing PC cell growth, invasiveness, and migration. However, the mechanism underlying LNC01094’s regulatory effects on PC cell functions needs further investigation.

## Other malignancies

In addition to the abovementioned types of cancers, LINC01094 was also observed to be significantly overexpressed in other malignancies, including colorectal cancer (CRC) (Zhang et al., 2022), hepatocellular carcinoma (HCC) (Yang et al., 2024), breast cancer (BC) (Wu, Kong & Wu, 2021), and laryngeal squamous cell carcinoma (LSCC) (Qian et al., 2022). Especially in CRC patients, high LINC01094 expression exhibited close correlations with patient vascular invasion, lymph node metastasis, and TNM classification (Zhang et al., 2022). Additionally, functioning as a competitive endogenous RNA, LINC01094 sponged miR-122-5p to activate the TGFBR2-SMAD2-SMAD3 axis and enhance HCC cell migration and invasion, while in breast cancer, it promoted proliferation, cell cycle progression, and lung metastasis by sequestering miR-342-5p and upregulating E2F3 (Wu, Kong & Wu, 2021; Yang et al., 2024). Moreover, Qian et al. (2022) showed that LINC01094/LINC02154, one of the seven immune-related lncRNA pairs, may be associated with the prognosis of LSCC patients, a finding that will provide guidance for clinicians regarding medication (Qian et al., 2022). In conclusion, LINC01094 is tightly associated with cancer occurrence and development and may represent a new marker or therapeutic target for the diagnosis and treatment of malignancies.

## Biological functions of LINC01094 and its regulatory mechanisms in cancers

### PTEN/PI3K/AKT pathway

**Figure 3:**
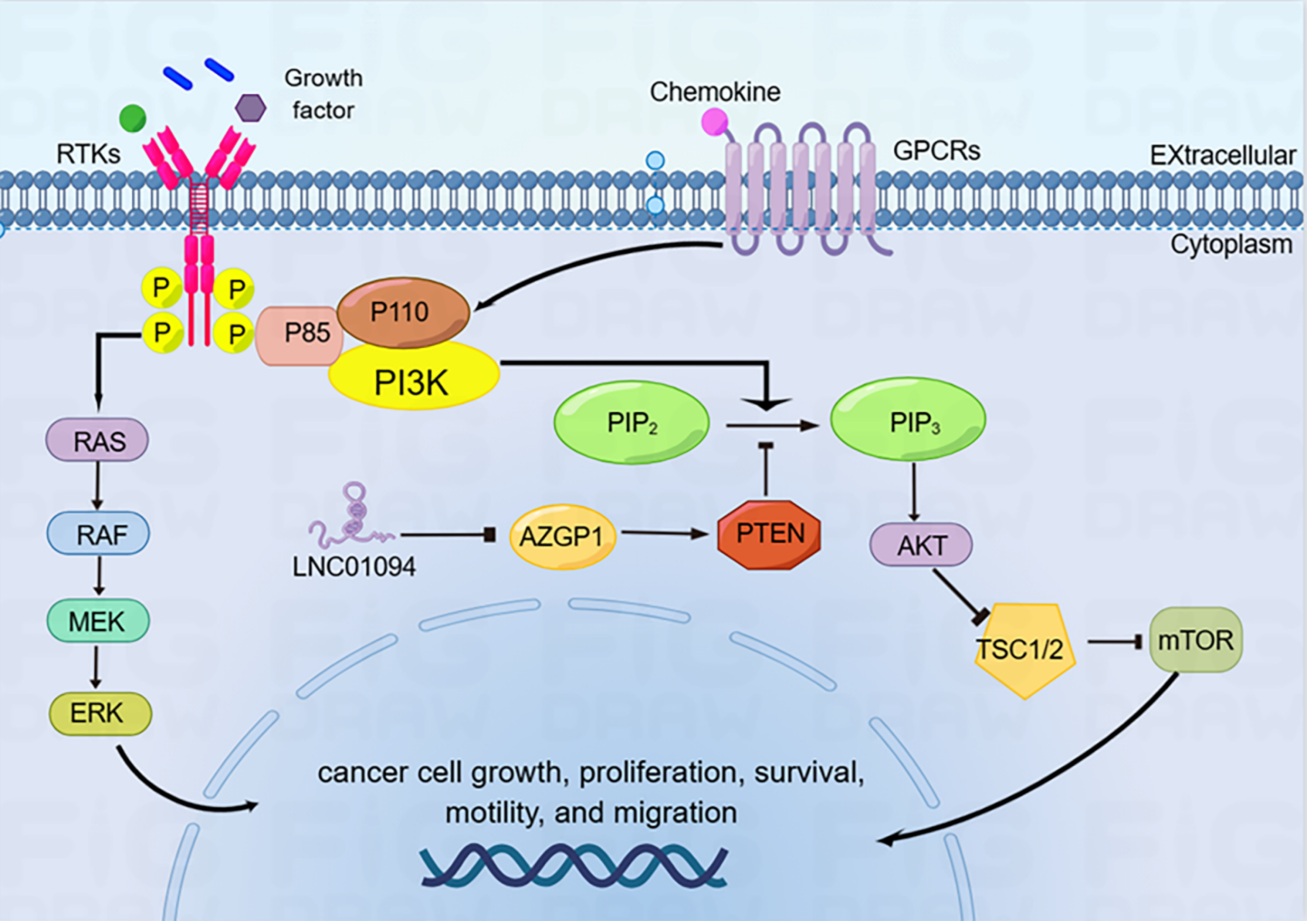
LINC01094 functionally modulates the PTEN/PI3K/AKT oncogenic signaling axis. LINC01094 induces cancer cell growth, proliferation, survival and migration through the activation of PTEN/PI3K/AKT signaling pathways.

PTEN/PI3K/AKT is a crucial signaling pathway involved in regulating various biological processes, including growth, proliferation, metabolism, and apoptosis, in a variety of cancers (Cao et al., 2020; Qian et al., 2022). PTEN is an essential suppressor for various primary and metastatic human cancers. It can prevent tumor initiation and progression by effectively antagonizing the PI3K/AKT signaling cascade (Hu et al., 2019). It has been recently revealed that LINC01094 boosted GC cell migration and proliferation by antagonizing AZGP1 function and thereby inhibiting the PTEN/AKT pathway (Gong et al., 2023). Conversely, down-regulation of LINC01094 can suppress the PI3K/AKT cascade by targeting miR-577, and subsequently inhibit the tumorigenesis and metastasis of pancreatic cancer (Luo et al., 2021). Collectively, these aforementioned observations suggest that LINC01094 functioning as a crucial molecule through its role in downregulating PTEN to activate PI3K/AKT (Fig. 3).

### Wnt/β-catenin signaling pathway

**Figure 4:**
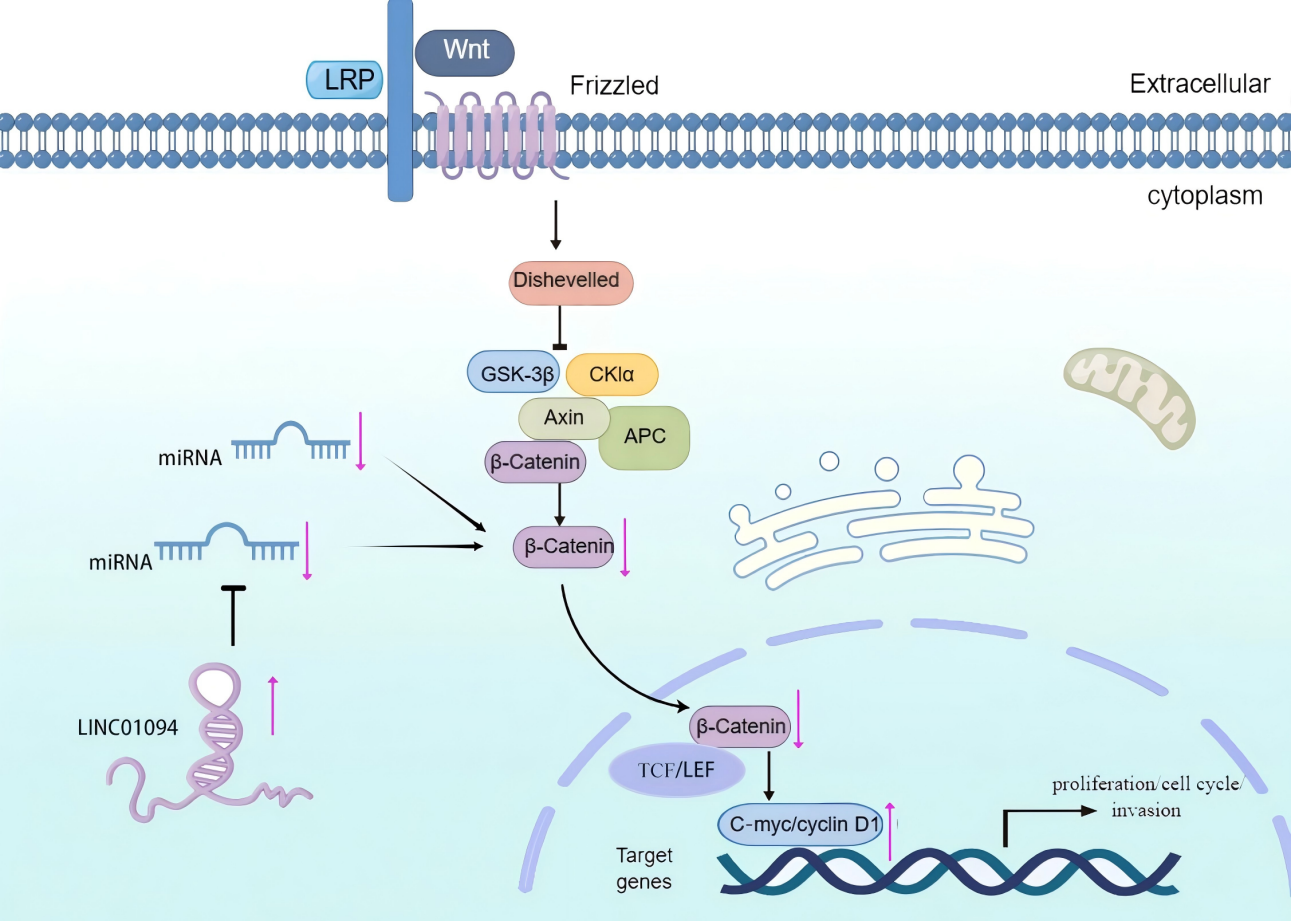
The role of LINC01094 in the Wnt/β-catenin signaling pathway. LINC01094 induces tumorigenesis and promotes tumor progression through the activation of Wnt/β-catenin signaling pathways.

It is generally accepted that the classical Wnt signaling pathway is a crucial regulator for tumor cell differentiation and proliferation. Notably, the core protein β-catenin of this pathway (Zhang & Wang, 2020) can mediate the transmission of signals from the cytoplasm to the nucleus, participate in the formation of transcription factor complexes, and stimulate the transcription of downstream target genes (Nusse & Clevers, 2017). Existing studies have shown that C-myc and cyclin D1, which are involved in the proliferation, circulation, and invasion of tumor cells, are downstream targets of β-catenin (Li & Ji, 2003). Interestingly, it has been demonstrated that LINC01094 can enhance the EMT, growth, invasiveness, and migration of tumor cells by targeting miR-577 and stimulating the Wnt/β-catenin cascade (Xu et al., 2020b). Therefore, the Wnt/β-catenin signaling pathway may be implicated with the role of LINC01094 in promoting tumor initiation and progression (Fig. 4).

## LINC01094/miRNA/axis

**Figure 5:**
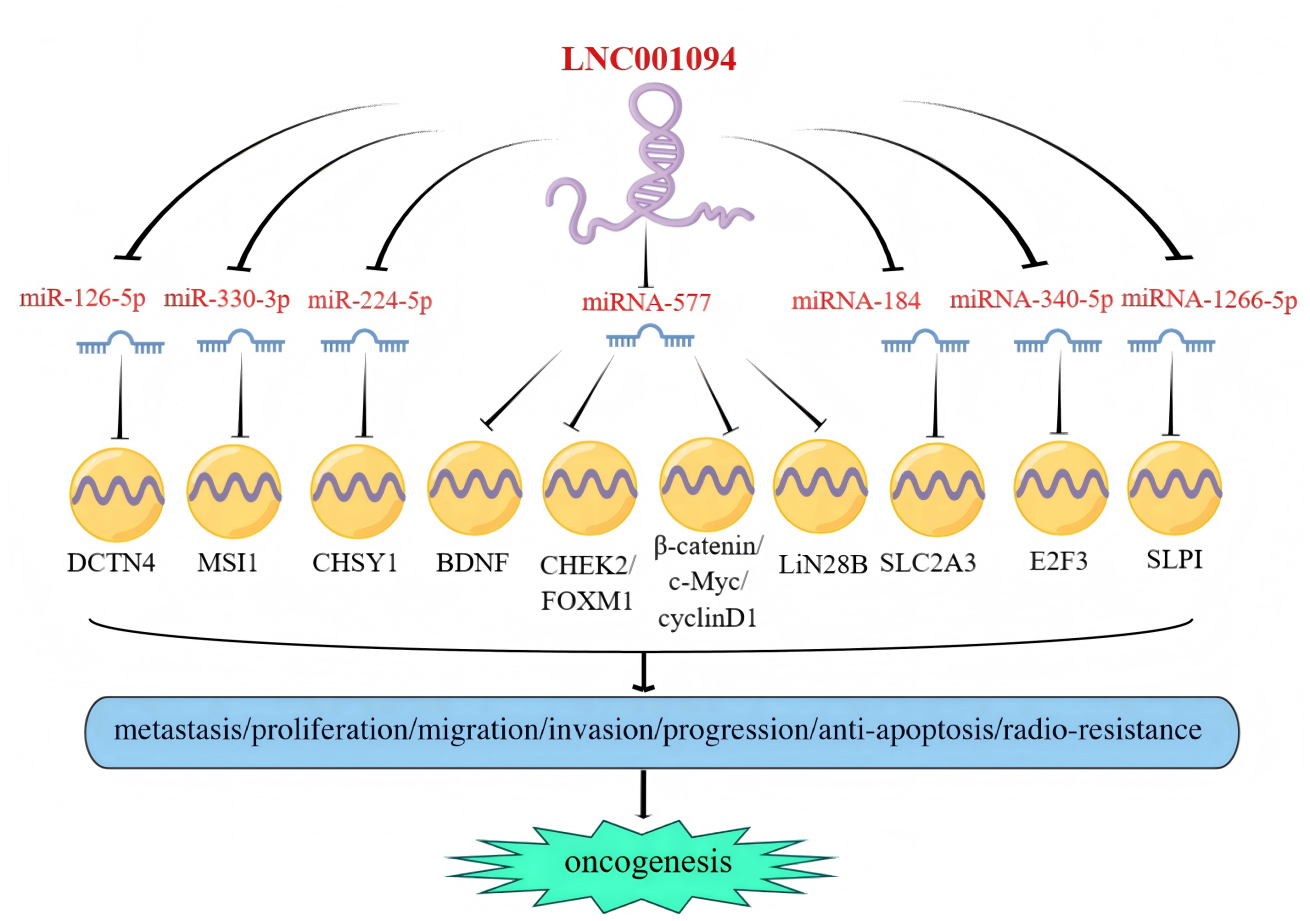
LINC01094 drives tumorigenesis *via* ceRNA-dependent miRNA sponging. LINC01094 acts as a ceRNA in multiple human cancers and exerts oncogenic effects by sponging miRs and directly reversing their suppression of tumorigenesis.

In recent years, the competitive endogenous RNA (ceRNA) hypothesis has been increasingly utilized to study mechanisms underlying cancer formation and development (Wei et al., 2017). As a ceRNA, LINC01094 was found to exert multiple molecular functions by interacting with downstream target miRNAs. The canonical mechanisms of miRNA action include translational repression and/or mRNA destabilization *via* 3′ UTR binding (Qi et al., 2015) to negatively regulate target gene expression. In 2021, Luo et al. (2021) found that LINC01094 promoted the expression of LIN28B in PC cells by sponging miR-577, a negative regulator of LIN28B, and facilitated the metastasis and proliferation of the tumors. Furthermore, LINC01094 could also serve as a ceRNA to competitively bind miR-340-5p, which resulted in the proliferation and cell cycle progression of BC cells (Wu, Kong & Wu, 2021). Additionally, the upregulation of LINC01094 was shown to enhance the progression of GBM by sponging miR-126-5p (Li & Yu, 2020), miR-330-3p (Zhu et al., 2020), and miR-577 (Luo et al., 2021). In ccRCC, reports have revealed that LINC01094 can sponge miR-224-5p, miR-577, and miR-184 to promote tumor development (Jiang et al., 2020a; Jiang et al., 2020b; Xu et al., 2020a). Moreover, LINC01094 was also found to closely related to the development and prognosis of other malignancies, such as LSCC (Sun et al., 2022) and GC (Gong et al., 2023), by functioning as a ceRNA. Furthermore, our target prediction analysis using the lncRNASNP2 database (https://guolab.wchscu.cn/lncRNASNP) revealed that miR-139, miR-195, and miR-200 may serve as potential target miRNAs of LINC01094. Taken together, LINC01094 may competitively bind its target miRNAs *via* the ceRNA mechanism, enhance the expression of downstream mRNAs, and subsequently facilitate tumor progression (Fig. 5).

## Conclusions

In the past 10 years, lncRNAs have become a hotspot in the field of cancer pathogenesis research. Among the lncRNAs investigated, LINC01094 is a newly discovered one and was found to play pivotal roles in tumor development and prognosis. In this review, we have summarized the roles of LINC01094 in the occurrence and development of multiple cancers. Numerous investigations showed that LINC01094 is an oncogenic lncRNA that is upregulated in various cancers and related to key cancer characteristics such as histological grade, tumor size, TNM staging, lymph node metastasis, and overall survival rate. The expression level of LINC01094 was found to be tightly correlated with the occurrence and development of multiple malignancies. In addition, down-regulation of LINC01094 was demonstrated to suppress the growth, invasiveness, and migrating capability of various types of cancerous cells *in vivo* and *in vitro*, implying that LINC01094 may potentially become a target in cancer management.

It is worth noting that the molecular function of LINC01094 is related to its subcellular localization. When localized in the cytoplasm, LINC01094 can act as a ceRNA to regulate tumor cell growth, invasion, and migration through signaling cascades such as PI3K/AKT, PTEN/AKT, and Wnt/β-catenin, and play carcinogenic roles in various cancer *via* transcriptional regulation mechanisms. When localized in the nucleus, LINC01094 could promote the nuclear translocation of SPI1, which further activates the transcription of CCL7 and ultimately leads to M2 macrophage accumulation and cancer cell dissemination (Wu et al., 2022). However, whether nuclear LINC01094 plays other roles in cancer cells requires further investigation.

Although the effects and underlying mechanisms of LINC01094 in regulating tumor cell functions have already been elucidated, the potential value of LINC01094 in evaluating the initiation, progression, and prognosis of various cancers warrants further exploration. More importantly, given the identified pathogenic functions of LINC01094 in various malignancies, identifying LINC01094 inhibitors and assessing their anticancer effects may provide new therapeutic drugs for cancer treatment. In summary, in light of the biological significance of LINC01094, its physiological and pathogenic functions, as well as its clinical applicability, should be further explored in the future.

##  Supplemental Information

10.7717/peerj.20621/supp-1 Supplemental Information 1 Study selection flow This diagram summarizes the systematic search in PubMed, EMBASE and Web of Science (2000–2025) employing keywords including LINC01094, lncRNA carcinoma and cancer, followed by sequential title/abstract and full-text screening to identify eligible studies for analysis.

10.7717/peerj.20621/supp-2 Supplemental Information 2 MiRNA target prediction MiRNA target prediction for LINC01094 was performed using the lncRNASNP2 database ( http://bioinfo.life.hust.edu.cn/lncRNASNP2).

## Additional Information and Declarations

### Data Availability

The following information was supplied regarding data availability: The raw data is available in the Supplementary File.
