# Supplementary figures and images for: Implications of LINC01094 for human malignancies
Source: PeerJ. 2026 Jan 27;14:e20621. doi: 10.7717/peerj.20621 (PMC12857556; doi:10.7717/peerj.20621)

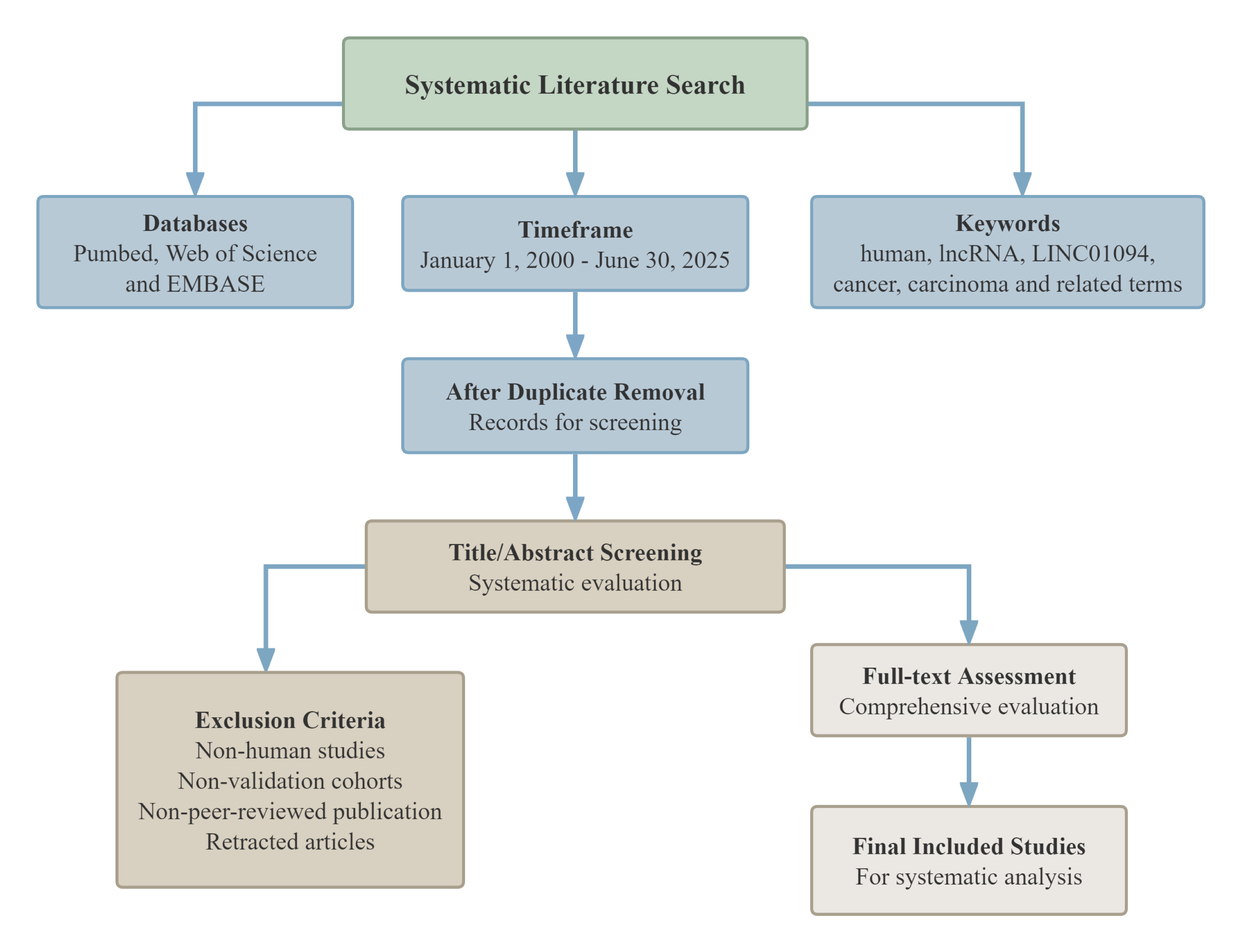

Supplement: Supplemental Information 1 — This diagram summarizes the systematic search in PubMed, EMBASE and Web of Science (2000–2025) employing keywords including LINC01094, lncRNA carcinoma and cancer, followed by sequential title/abstract and full-text screening to identify eligible studies for analysis. [file peerj-14-20621-s001.png]
